# Dihydromyricetin ameliorates osteogenic differentiation of human aortic valve interstitial cells by targeting c-KIT/interleukin-6 signaling pathway

**DOI:** 10.3389/fphar.2022.932092

**Published:** 2022-08-08

**Authors:** Shaoshao Zhang, Leilei Fan, Yongjun Wang, Jianjun Xu, Qiang Shen, Jianhua Xie, Zhipeng Zeng, Tingwen Zhou

**Affiliations:** ^1^ Department of Cardiovascular Surgery, Union Hospital, Tongji Medical College, Huazhong University of Science and Technology, Wuhan, China; ^2^ Department of Cardiology, Union Hospital, Tongji Medical College, Huazhong University of Science and Technology, Wuhan, China; ^3^ Department of Gastrointestinal Surgery, Central Hospital of Enshi Tujia and Miao Autonomous Prefecture, Enshi, China; ^4^ Department of Hepatobiliary Surgery, Union Hospital, Tongji Medical College, Huazhong University of Science and Technology, Wuhan, China; ^5^ Department of Rheumatology and Immunology, Tongji Hospital, Tongji Medical College, Huazhong University of Science and Technology, Wuhan, China

**Keywords:** dihydromyricetin, interleukin-6, c-kit, human valvular interstitial cells, calcific aortic valve disease dihydromyricetin ameliorates aortic valve calcification

## Abstract

**Aims:** Calcific aortic valve disease (CAVD) is a chronic cardiovascular disease with high morbidity that lacks effective pharmacotherapeutics. As a natural flavonoid extracted from Ampelopsis grossedentata, dihydromyricetin (DHM) has been shown to be effective in protecting against atherosclerosis; yet, the therapeutic role of DHM in CAVD remains poorly understood. Herein, we aimed to clarify the therapeutic implications of DHM in CAVD and the underlying molecular mechanisms in human valvular interstitial cells (hVICs).

**Methods and Results:** The protein levels of two known osteogenesis-specific genes (alkaline phosphatase, ALP; runt-related transcription factor 2, Runx2) and calcified nodule formation in hVICs were detected by Western blot and Alizarin Red staining, respectively. The results showed that DHM markedly ameliorated osteogenic induction medium (OM)–induced osteogenic differentiation of hVICs, as evidenced by downregulation of ALP and Runx2 expression and decreased calcium deposition. The SwissTargetPrediction database was used to identify the potential AVC-associated direct protein target of DHM. Protein–protein interaction (PPI) analysis revealed that c-KIT, a tyrosine-protein kinase, can act as a credible protein target of DHM, as evidenced by molecular docking. Mechanistically, DHM-mediated inhibition of c-KIT phosphorylation drove interleukin-6 (IL-6) downregulation in CAVD, thereby ameliorating OM-induced osteogenic differentiation of hVICs and aortic valve calcification progression.

**Conclusion:** DHM ameliorates osteogenic differentiation of hVICs by blocking the phosphorylation of c-KIT, thus reducing IL-6 expression in CAVD. DHM could be a viable therapeutic supplement to impede CAVD.

## Introduction

Calcific aortic valve disease (CAVD) is an irreversible disease associated with severe aortic valve stenosis and is a major contributor to mortality in cardiac patients (Kraler et al., 2022). Despite mounting evidence proving a crucial role for lipoprotein (a) and low-density lipoprotein cholesterol in the progression of CAVD (Mundal et al., 2019; Chen et al., 2020; Kaltoft et al., 2022), lipid-lowering has failed to blunt CAVD progression (Chan et al., 2010; Teo et al., 2011). Thus, transaortic and surgical valvular replacement remains the most effective treatments for CAVD, and there is an urgent need to explore novel disease-modifying pharmacotherapies for nonsurgical CAVD treatment. Human aortic valve interstitial cells (hVICs) play a pivotal role in mediating aortic valve calcification (AVC) by switching from quiescent hVICs to an osteoblast-like phenotype (Peeters et al., 2018; Issa et al., 2019). This phenotypic switch is identified as a prominent hallmark and driving factor of accelerated AVC (Wang et al., 2020; Wang Y et al., 2021). Thus, a deeper understanding of the pathophysiology of phenotypic transformation in hVICs is critical for providing novel therapeutic options for CAVD.

Increasing evidence shows that traditional Chinese herbal medicines have protective and curative pharmacological effects on cardiovascular diseases (Zhao et al., 2020; Zhang et al., 2021a; Zhang et al., 2021b; Zhu et al., 2021). Ampelopsis grossedentata (also known as vine tea) is a traditional Chinese edible herb widely consumed not only as a healthy tea but also as a medicinal herb that possesses numerous pharmacological activities. Dihydromyricetin (DHM), the most bioactive constituent of ampelopsis grossedentata, has increasingly drawn attention as an effective drug for the treatment of cardiovascular diseases, including vascular calcification (Feng et al., 2021), myocardial hypertrophy (Chen et al., 2018), doxorubicin-induced cardiotoxicity (Sun et al., 2020), and cardiac ischemia/reperfusion injury (Wei et al., 2019). Specifically, Yang et al. reported that DHM could ameliorate the progression of atherosclerosis in low-density lipoprotein receptor-deficient (LDLr^−/−^) mice by countering hyperlipidemia and aortic inflammation (Liu et al., 2017). A more recent study also demonstrated that DHM ameliorates atherosclerotic lesion formation by increasing endothelial nitric oxide production in apolipoprotein E-deficient mice (Yang et al., 2020). Importantly, numerous experimental and clinical studies suggest that CAVD and atherosclerosis share similar pathological features (Hutcheson et al., 2014; Cho et al., 2018). Thus, these findings suggest that DHM might also have a pharmacological effect on CAVD.

C-KIT (also known as CD117) is an oncogene belonging to the family of receptor kinases that is expressed in various cell types and tissues (Mukhopadhyay et al., 2011). Upon activation *via* its ligand, stem cell factor (SCF), c-KIT can activate several signaling pathways and thereby play a regulatory role in cardiovascular diseases, including vascular diseases (Kim et al., 2014; Hernandez et al., 2019), cardioprotection (Ebeid et al., 2020), and cardiac stem cell migration (Kuang et al., 2008). Moreover, given its biological properties, c-KIT has been widely recognized as a biomarker to identify presumptive cardiac stem cells that respond to myocardial injury (Gude et al., 2018) and hypertrophic cardiomyopathy (Sonnenschein et al., 2021). Furthermore, c-KIT-positive progenitor cells have been observed in pathological human cardiac valves (Veinot et al., 2006; Gendron et al., 2021). Nevertheless, the regulatory role of c-KIT in CAVD progression has not been experimentally investigated.

In the present study, the molecular docking results showed that DHM successfully formed hydrogen bonds with c-KIT with a docking binding energy of −10.3 kcal/mol. Moreover, the osteogenic induction medium (OM) significantly enhances the phosphorylation of c-KIT, thereby promoting osteogenic differentiation of hVICs, while DHM can markedly ameliorate the osteogenic differentiation of hVICs by inhibiting the phosphorylation of c-KIT. Importantly, the present data also indicated that IL-6 is a downstream target of c-KIT, contributing to DHM-mediated inhibition of the osteogenic differentiation of hVICs. Hence, DHM may represent a novel pharmacological supplement for preventing CAVD progression.

## Materials and methods

### Cell culture and treatment

Primary hVICs were isolated from human aortic valves as we previously described (Zhou et al., 2020; Wang Y et al., 2021). First, to remove valvular endothelial cells, noncalcified aortic valve leaflets were digested in 1 mg/ml type I collagenase (Sigma–Aldrich, Saint Louis, MO) for 30 min. Subsequently, the tissues were further digested in 2 mg/ml type I collagenase at 37°C in 5% CO_2_ for 8 h. Then, isolated hVICs were cultured in Dulbecco′s modified Eagle′s medium (DMEM, Gibco, Invitrogen Corporation, United States) containing 10% fetal bovine serum, 100 μg/ml streptomycin, and 100 U/ml penicillin with 5% CO_2_ at 37°C. Osteogenic induction medium (DMEM supplemented with 0.1% fetal bovine serum, 5 mmol/L β-glycerophosphate, 50 ng/ml ascorbic acid, 50 ng/ml BMP-2, and 100 nmol/L dexamethasone) was used to establish the osteogenic differentiation model of hVICs as previously described (Wang et al., 2020; Zhou et al., 2021). The treatment groups included the control (10% DMEM-treated group), OM (OM-treated group), and OM plus DHM (Selleck, Cat. No. S2399; 20 μM) (OM + DHM-treated group). ISCK03 (Selleck, Cat. No. S2070; at 5 μM final concentration in OM) was used to specifically inhibit c-KIT activity. Stem cell factor (Sigma–Aldrich, Catalog# H8416; at 50 ng/ml final concentration in OM) was used to induce phosphorylation of c-KIT. IL-6 (Sigma–Aldrich, Catalog# I1395; at 50 ng/ml final concentration in OM) was used to explore the underlying mechanism of the DHM-mediated inhibitory effect on the osteogenic differentiation of hVICs. The treatment groups included the OM group, IL-6+OM group, and IL-6+OM + DHM group. The medium was changed every 3 days.

### Cell viability analysis

To evaluate cell viability, a CCK-8 (K1018, APEXBIO) assay was carried out according to the manufacturer’s instructions as previously described (Zhou et al., 2020). First, primary hVICs were seeded in 48-well plates and incubated for 12 h in DMEM supplemented with 10% fetal bovine serum (FBS). Subsequently, the cells were further cultured in low-serum medium (2% FBS) for 24 h and then treated with different concentrations of DHM for 72 h to determine the half-maximal inhibitory concentration (IC_50_) values of DHM in hVICs. Furthermore, after treatment with 20 μM DHM for 6 days, cell viability was also measured. In brief, cells were washed with phosphate-buffered saline (PBS) and subsequently incubated with serum-free medium that contained 10% CCK-8 reagents for 2 h. Finally, light absorption at 450 nm was measured using an enzyme labeling instrument.

### Quantitative real-time polymerase chain reaction analysis

qRT–PCR analysis was performed as previously described (Xu et al., 2019; Wang Y et al., 2021). In brief, after treatment with the indicated medium, total RNA was isolated using TRIzol reagent (Invitrogen). The PrimeScript RT Reagent Kit (TaKaRa Bio, Otsu, Shiga, Japan) was used to synthesize cDNA according to the manufacturer’s instructions. Subsequently, qRT–PCR was conducted by a Step One Real-Time PCR System (Applied Biosystems, Foster City, CA, United States) using SYBR Green PCR reagent (TaKaRa). The data were normalized relative to glyceraldehyde-6-phosphate dehydrogenase (GAPDH) and expressed as a relative ratio using the -2^ΔΔCt^ method. The following primer sequences were used in the present study: ALP (forward: 5′-CGC​TGT​GTC​AAC​TCC​ACC​T-3′; reverse:5′-CCAGAAGGTTCTGTTAACTTG-3′); RUNX2 (forward: 5′-GCG​TCA​ACA​CCA​TCA​TTC​TG-3′; reverse:5′-CAGACCAGCAGCACTCCATC-3′); IL-6 (forward: 5′-TGG​CTG​CAG​GAC​ATG​ACA​ACT-3′; reverse:5′-ATCTGAGGTGCCCATGCTACA-3′); GAPDH (forward:5′-CCTCAAGAT CATCAGCAAT-3′; reverse: 5′-CCA​TCC​ACA​GTC​TTC​TGG​GT-3′).

### Western blotting assay

Western blotting assays were conducted as previously described (Wang Y et al., 2021; Zhou et al., 2021). In brief, after treatment with the indicated medium, total proteins were extracted from the hVICs using radioimmunoprecipitation assay (RIPA) buffer. The concentration of the protein lysate was calculated with the bicinchoninic acid (BCA) protein assay kit (Beyotime, Shanghai, China). Subsequently, equal protein extracts (30 μg/well) from each group were separated by 4–20% sodium dodecyl sulfate–polyacrylamide gel electrophoresis (SDS–PAGE) and then transferred to polyvinylidene fluoride (PVDF) membranes (Millipore, Billerica, MA, United States) in the presence of methanol. Next, the membranes were blocked with 5% nonfat milk in TBST and incubated with primary antibodies against ALP (1:1,000, 11187-1-AP, Proteintech), RUNX2 (1:1,000, #12556, Cell Signaling Technology), GAPDH (1:1,000, 60004-1-Ig, Proteintech), and phospho-c-Kit (Tyr703, 1:1,000, #3073, Cell Signaling Technology). Phospho-c-Kit (Tyr823, 1:1,000, #77522, Cell Signaling Technology), Phospho-c-Kit (Tyr721, 1:1,000, 44-494G, Invitrogen), Phospho-c-Kit (Tyr568, 1:1,000, #48347, Cell Signaling Technology), Phospho-c-Kit (Tyr719, 1:1,000, #3391, Cell Signaling Technology), c-KIT (1:1,000, 34-8800, Invitrogen), and IL-6 (1:1,000, P620, Invitrogen) at 4 °C. Finally, the membranes were specifically incubated with the HRP-conjugated secondary antibody for 2 h at room temperature and detected by chemiluminescence using a Western blot imaging system (Clinx Science Instruments, Shanghai, China). GAPDH acted as an internal reference, and all data were analyzed by ImageJ 1.55 software (National Institutes of Health).

### Alizarin red staining

The Alizarin Red staining method was used to visualize calcified nodule formation in hVICs as previously described (Xu et al., 2018; Wang et al., 2020). First, after the indicated treatments, hVICs from each group were rinsed in 1×PBS three times and fixed in 4% paraformaldehyde (PFA) for 15 min at room temperature. After that, the cells were washed and exposed to 2% Alizarin Red stain (Sigma–Aldrich) for 15 min according to the manufacturer’s instructions. Subsequently, after washing three times with deionized water to remove excess Alizarin Red dye, images were visualized using an Olympus BX51 microscope. The red staining (arbitrary units shown) indicated calcified nodule formation. The quantification of Alizarin Red staining was performed as we described previously (Wang Y et al., 2021). After obtaining high-resolution images, Adobe Photoshop CC was used to edit the image contrast and brightness. Then, ImageJ 1.55 (National Institutes of Health) software was used to determine the positive stained area (arbitrary units shown) of Alizarin Red per magnified field, and the data were averaged for three independent biological replicates.

### Molecular target analysis

The potential protein targets of DHM were obtained from the SwissTargetPrediction database (http://www.swisstargetprediction.ch). The aortic valve calcification differentially expressed genes (AVCDEGs) were collected based on our previously uploaded RNA-seq data in the NCBI SRA database at PRJNA643215 and PRJNA552159. After data extraction, a comprehensive bioinformatic analysis of the top 10 candidate targets and 216 AVCDEGs was performed by protein–protein interaction (PPI) analysis according to STRING (https://www.string-db.org/) with Cytoscape software (Wang C et al., 2021). The potential AVC-associated molecular target of DHM was selected, and c-KIT with the highest degree at a confidence value of 0.4 was chosen as a potential DHM molecular target for further analysis.

### Molecular docking

Molecular docking was performed as previously described (Ye et al., 2019). The compound name, molecular weight, and three-dimensional (3D) structure of DHM were obtained from the PUBCHEM database. The 3D structure of c-KIT was obtained from the Protein Data Bank (PDB, ID: 1 pkg) database. Subsequently, AutoDock Vina software (http://vina.scripps.edu/) was used to prepare the ligands and proteins required for molecular docking. Finally, the docking results were analyzed by Discovery Studio software 2019 (DS 2019). The combination capability of DHM and c-KIT was evaluated by the affinity (kcal/mol) value.

### Immunofluorescence staining

Immunofluorescence staining was applied for the detection of ALP, RUNX2, phospho-c-Kit (Tyr703), phospho-c-Kit (Tyr721), and IL-6 in hVICs as previously described (Dai et al., 2019; Zhou et al., 2021). Briefly, following the indicated treatments, hVICs were rinsed in 1× PBS and then fixed in 4% PFA for 20 min. Subsequently, the fixed cells were permeabilized with 0.1% Triton X-100 in PBS for another 20 min and incubated with the following primary antibodies at 4°C overnight: ALP (1:200, 11187-1-AP, Proteintech), RUNX2 (1:200, #12556, Cell Signaling Technology), phospho-c-Kit (Tyr703, 1:200, 710762, Invitrogen), phospho-c-Kit (Tyr721, 1:200, 44-494G, Invitrogen), and IL-6 (1:200, P620, Invitrogen). Finally, followed by incubation with fluorescently conjugated secondary antibody (Abcam, Cambridge, MA, United States) and counterstaining with 4′,6‐diamidino‐2‐phenylindole (DAPI) (Sigma–Aldrich), the cells were then visualized by a confocal laser scanning microscope FV3000 (Olympus, GmbH, Hamburg, Germany).

### Statistical analysis

All values are presented as the mean ± standard deviation (SD) and were analyzed using GraphPad Prism 8 software (GraphPad Software, Inc., CA, United States). For continuous data with a normal distribution and equal variances, Student’s *t* test was applied between two groups and one-way analysis of variance (one-way ANOVA) followed by Bonferroni multiple comparison *post hoc test* was performed for multiple comparisons (≥3 groups). All semiquantitative measurements were performed by ImageJ 1.55 (National Institutes of Health) software. *p* < 0.05 was considered statistically significant.

## Results

### DHM inhibits osteogenic differentiation of hVICs *in vitro*

**FIGURE 1:**
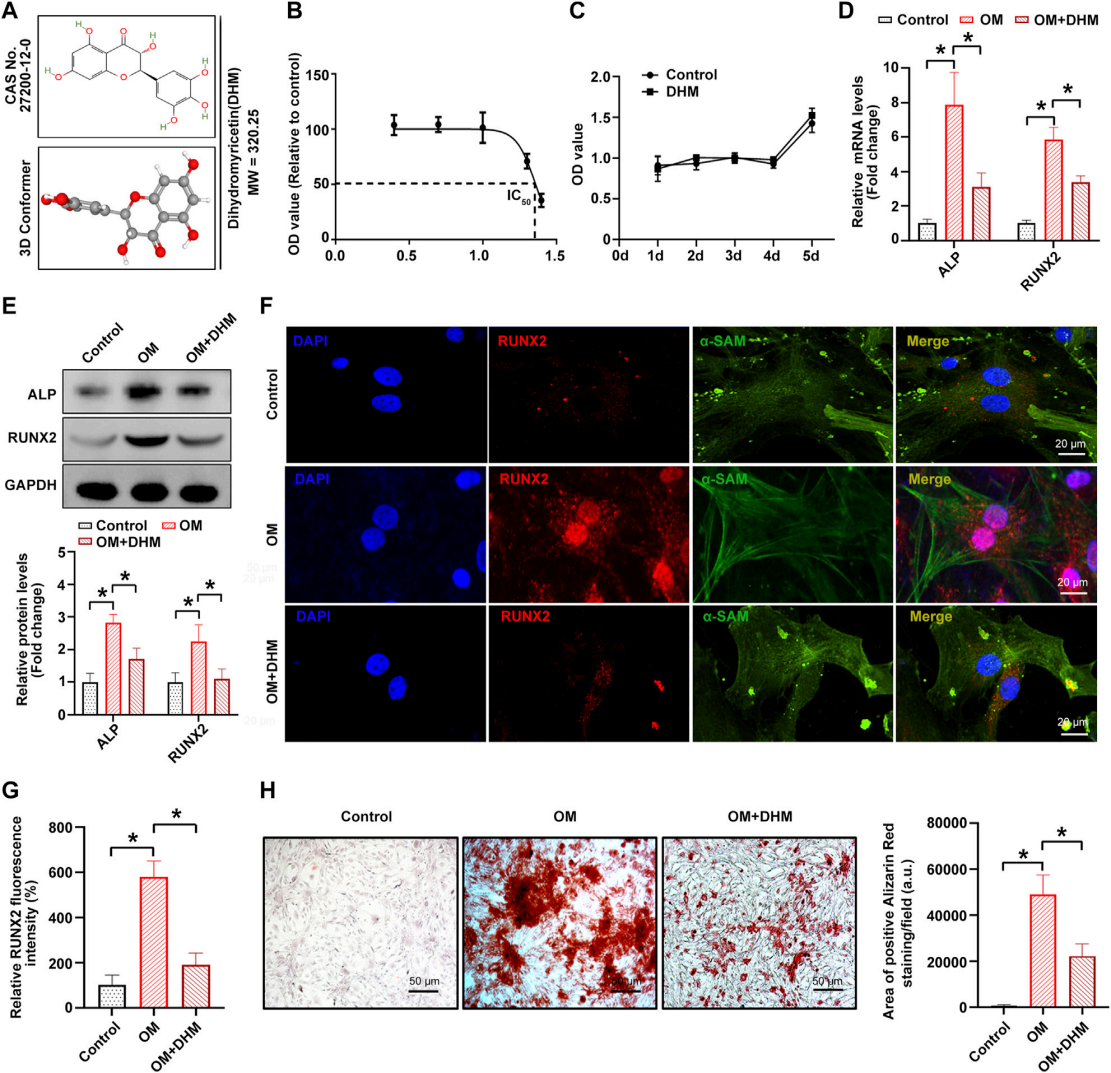
Dihydromyricetin (DHM) inhibits osteogenic differentiation of human valvular interstitial cells (hVICs). **(A)** Molecular structure of DHM. **(B)** Half-maximal inhibitory concentration (IC_50_) of DHM on hVICs; concentrations were transferred to Log**(C)**. **(C)** Cell Counting Kit-8 (CCK-8) assay was conducted to detect the cell proliferation rate of hVICs with the indicated treatment for 5 days. The mRNA **(D)** and protein **(E)** levels of osteogenesis-specific genes (alkaline phosphatase, ALP, and runt-related transcription factor 2, RUNX2) in hVICs stimulated with osteogenic induction medium (OM) and then treated with or without DHM. One-way ANOVA followed by Bonferroni *post hoc test*. Immunofluorescence staining **(F)** and semiquantification **(G)** of RUNX2 in hVICs stimulated with OM and then treated with or without DHM. One-way ANOVA followed by Bonferroni *post hoc test*. **(H)** Alizarin red staining of mineralization nodules in hVICs stimulated with OM and then treated with or without DHM. One-way ANOVA followed by a Bonferroni *post hoc test*. *N* = 3 per group. Values are the mean ± SD. *p < 0.05 indicates a significant difference.

Figure 1A shows the chemical formula and 3D molecular structure of DHM (molecular weight, 320.25 Da) obtained from the PubChem database. In the present study, the IC_50_ values of DHM-treated hVICs were approximately 20–30 μM (Figure 1B). Thus, a final DHM concentration of 20 μM was used for further experiments. Furthermore, the CCK-8 assay indicated that the viability of hIVCs did not differ from that of the control group after treatment with DHM for 5 days (Figure 1C). As the osteoblast-like phenotypic conversion of hVIC is a critical step in CAVD pathogenesis (Wang Y et al., 2021; Zhou et al., 2021), we investigated whether DHM orchestrated osteogenic differentiation of hVIC. To stimulate osteogenic differentiation, hVICs were exposed to OM for 14 days as previously described (Wang Y et al., 2021). Then, we explored the effects of DHM in hVICs and found that DHM treatment significantly negated the OM-induced increase in both the mRNA (Figure 1D) and protein (Figures 1E–G) levels of two known osteogenesis-specific genes (ALP and Runx2). Finally, DHM treatment significantly reduced the OM-induced increase in calcified nodule formation in hVICs (Figure 1H). These results suggest that DHM exhibits highly inhibitory effects on the osteogenic differentiation of hVICs.

### DHM targets c-KIT and inhibits its phosphorylation induced by OM in hVICs

**FIGURE 2:**
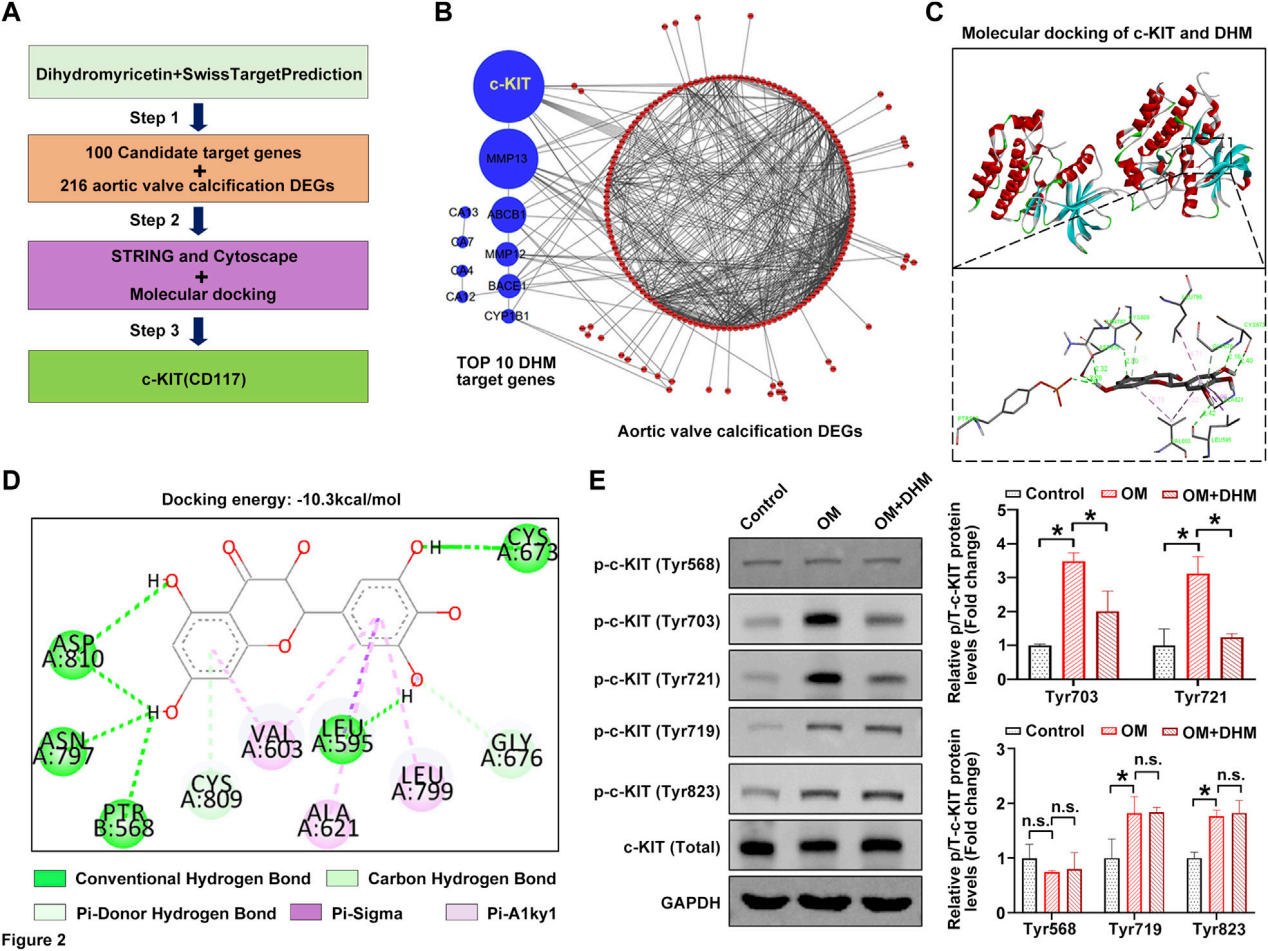
Dihydromyricetin (DHM) targets c-KIT involved in the regulation of aortic valve calcification (AVC). **(A)** Flowchart of the identification of AVC-associated target genes regulated by DHM. **(B)** Protein–protein interaction (PPI) network of the top 10 candidate targets of DHM and 216 AVC differentially expressed genes (DEGs). **(C)** Molecular docking of DHM and c-KIT. **(D)** Two-dimensional (2D) diagram of the binding position between DHM and c-KIT. **(E)** Western blotting was used to detect the phosphorylation levels of c-KIT in human valvular interstitial cells (hVICs) stimulated with osteogenic induction medium (OM) and then treated with or without DHM. One-way ANOVA followed by Bonferroni *post hoc test*. *N* = 3 per group. Values are the mean ± SD. *p < 0.05 indicates a significant difference. The n. s. indicates no significant difference.

We next used the online tool SwissTargetPrediction to further investigate the precise mechanism by which osteogenic differentiation of hVICs is regulated by DHM. Figure 2A shows the flowchart of the identification of AVC-associated target genes regulated by DHM. First, 100 potential target proteins of DHM were identified by SwissTargetPrediction. Then, a comprehensive bioinformatic analysis of the top 10 candidate targets and 216 AVC differentially expressed genes (AVCDEGs) was performed to identify target proteins underlying the effect of DHM on CAVD. The AVCDEGs were obtained from our previous upload to the NCBI SRA database at PRJNA643215 and PRJNA552159 (Xu et al., 2020). Subsequently, the protein–protein interaction (PPI) network of the top 10 candidate targets and AVCDEGs was generated by the STRING database (https://www.string-db.org/) with Cytoscape software, which found c-KIT with the highest degree at a confidence value of 0.4 (Figure 2B). To identify the possible modes and sites of c-KIT responsible for DHM binding, we performed molecular docking using AutoDock Vina software. The 3D docking mode and interaction details show that DHM successfully formed hydrogen bonds with c-KIT, with a docking binding energy of −10.3 kcal/mol (Figure 2C). As shown in Figure 2D, for the c-KIT-DHM complex, ASP810, ASN797, PTR568, LEU595, CYS673, CYS809, GLY676, VAL603, ALA621, and LEU799 formed some interactions with different moieties of DHM, which made the binding of c-KIT and DHM stable. Upon phosphorylation by its cytokine ligand, SCF, c-KIT can activate several signaling pathways (Kim et al., 2014). Thus, we explored whether its phosphorylation was altered following DHM treatment in hVICs. The results revealed that DHM significantly negated the OM-induced increase in the levels of phosphorylated c-Kit at sites Tyr703 and Tyr721 in hVICs (Figure 2E). These data suggest that DHM significantly reduced the OM-induced phosphorylation of c-KIT in hVICs.

### C-KIT phosphorylation inhibition alleviates the osteogenic differentiation of hVICs

**FIGURE 3:**
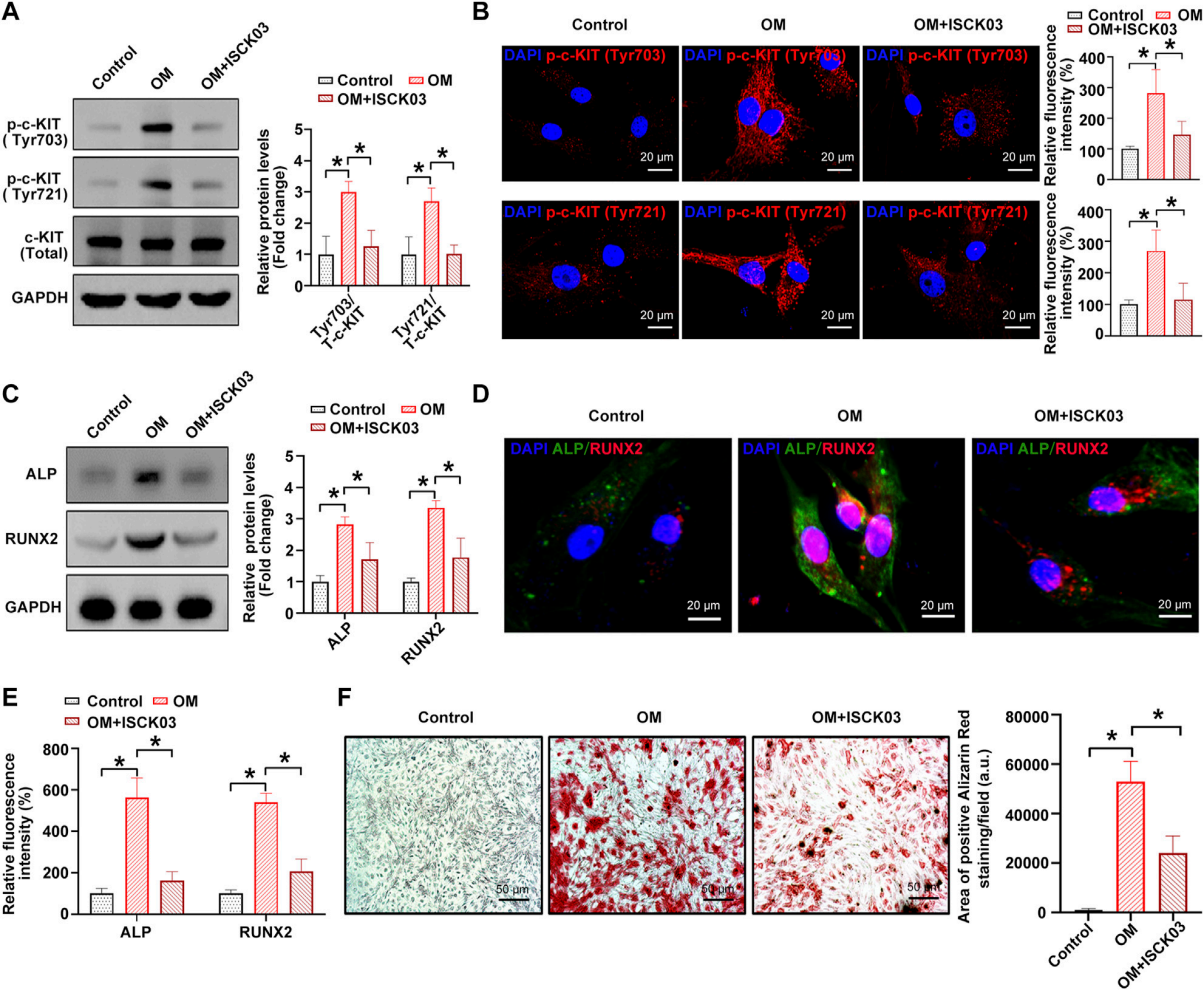
c-KIT inhibition prevents the osteogenic differentiation of human valvular interstitial cells (hVICs). Western blotting **(A)** and immunofluorescent staining **(B)** were used to confirm the inhibitory effect of ISCK03 (specific inhibitor of c-KIT) on c-KIT activity. One-way ANOVA followed by a Bonferroni *post hoc test*. Western blotting **(C)** and immunofluorescent staining **(D)** were used to detect the protein levels of osteogenesis-specific genes (alkaline phosphatase, ALP, and runt-related transcription factor 2, RUNX2) in human valvular interstitial cells (hVICs) stimulated with osteogenic induction medium (OM) and then treated with or without ISCK03. **(E)** Semiquantification of the fluorescence intensity of RUNX2 and ALP. One-way ANOVA followed by Bonferroni *post hoc test*. **(F)** Alizarin red staining of mineralization nodules in hVICs stimulated with OM and then treated with or without ISCK03. One-way ANOVA followed by Bonferroni *post hoc test*. *N* = 3 per group. Values are the mean ± SD. *p < 0.05 indicates a significant difference.

Next, [4-t-butylphenyl]-N-(4-imidazol-1-yl phenyl) sulfonamide (also known as ISCK03), a selective c-KIT kinase activity inhibitor, was used to inhibit the phosphorylation of c-KIT in hVICs, thereby confirming whether c-KIT plays a role in AVC. The results showed that OM-induced phosphorylation of c-Kit at sites Tyr703 and Tyr721 was successfully decreased by ISCK03, as evidenced by Western blotting (Figure 3A) and immunofluorescence (Figure 3B), respectively. Next, hVICs were exposed to OM for 14 days to stimulate osteogenic differentiation and then treated with ISCK03 (5 μM). The results indicated that ISCK03 significantly negated the OM-induced increase in the protein levels of two known osteogenesis-specific genes (ALP and Runx2), as evidenced by Western blotting (Figure 3C) and immunofluorescence (Figures 3D,E). Importantly, ISCK03 treatment significantly alleviated the OM-induced increase in calcified nodule formation in hVICs (Figure 3F). These results suggest that inhibition of c-KIT phosphorylation exhibits highly inhibitory effects on osteogenic differentiation of hVICs.

### DHM represses interleukin-6 through c-KIT inhibition in hVICs

**FIGURE 4:**
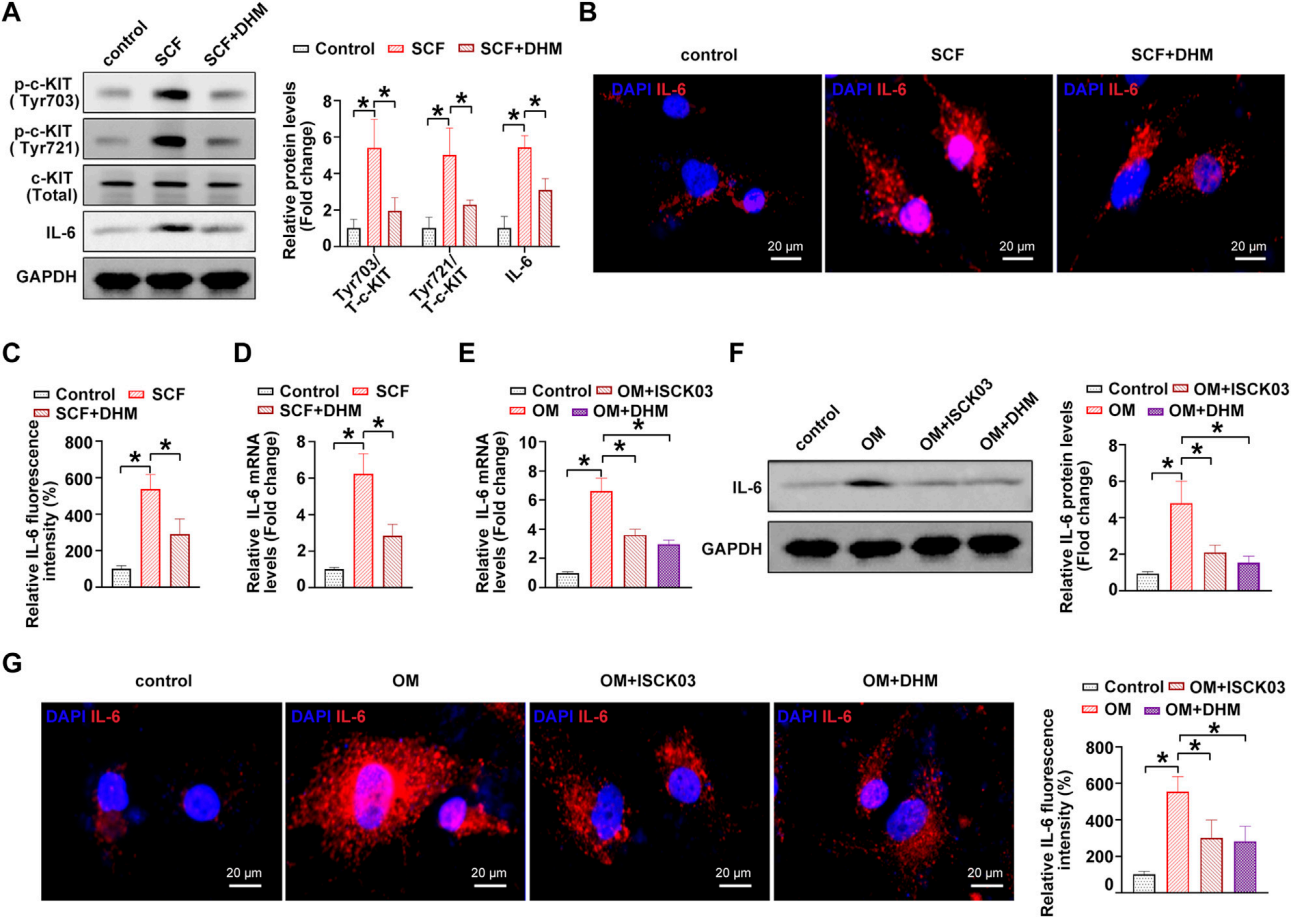
Dihydromyricetin (DHM) represses interleukin-6 (IL-6) through c-KIT inhibition in human valvular interstitial cells (hVICs). **(A)** IL-6 and phosphorylated c-Kit (sites of Tyr703 and Tyr721) protein levels in hVICs stimulated with stem cell factor (SCF, 100 ng/ml), the c-kit ligand, or cotreated with DHM. One-way ANOVA followed by Bonferroni *post hoc test*. **(B)** Immunofluorescent staining was used to detect the protein levels of IL-6 in hVICs following different conditioned culturing conditions. **(C)** Semiquantification of the fluorescence intensity of IL-6. One-way ANOVA followed by Bonferroni post hoc test. One-way ANOVA followed by Bonferroni *post hoc test*. **(D)** mRNA level of IL-6 in hVICs stimulated with SCF and then treated with or without DHM. One-way ANOVA followed by Bonferroni *post hoc test*. **(E)** IL-6 mRNA levels in hVICs stimulated with osteogenic induction medium (OM), or cotreated with ISCK03 or DHM. One-way ANOVA followed by Bonferroni *post hoc test*. **(F)** IL-6 protein levels in hVICs stimulated with OM or cotreated with ISCK03 or DHM. One-way ANOVA followed by Bonferroni *post hoc test*. **(G)** Immunofluorescent staining was used to detect the protein level of IL-6 in hVICs following different conditioned culturing conditions. One-way ANOVA followed by Bonferroni *post hoc test*. *N* = 3 per group. Values are the mean ± SD. *p < 0.05 indicates a significant difference.

A previous study showed that c-KIT is an important regulator of interleukin-6 (IL-6) secretion (Krishnamoorthy et al., 2008). The overactivation of c-KIT stimulates the secretion of IL-6, thereby inducing inflammation (Das Roy et al., 2013). In addition, IL-6 was experimentally shown to be a strong inducer of the mineralization of valve interstitial cells (El Husseini et al., 2013; El Husseini et al., 2014). Thus, we hypothesized that IL-6 might be the downstream target of c-KIT in the phenotypic switching of hVICs and thereby affect OM-induced AVC *in vitro*. To address this issue, we further explored the role of IL-6 in the DHM-mediated inhibitory effects on the osteogenic differentiation of hVICs. Western blot (Figure 4A), immunofluorescence (Figures 4B,C) and qRT–PCR (Figure 4D) results showed that IL-6 levels were significantly increased upon treatment with the c-kit ligand, SCF, while DHM treatment partially abolished the SCF-induced upregulation of IL-6, as expected. Moreover, IL-6 mRNA (Figure 4E) and protein (Figures 4F,G) levels were significantly increased upon OM stimulation, and inhibition of c-KIT with ISCK03 markedly reduced this effect in hVICs. Importantly, we also found that DHM was able to effectively reverse the OM-induced increases in IL-6 mRNA (Figure 4E) and protein levels (Figures 4F,G). These results suggest that DHM functions as a novel inhibitor of c-KIT, thereby decreasing IL-6 protein expression in hVICs.

### DHM inhibits the osteogenic differentiation of hVICs *via* IL-6 downregulation

**FIGURE 5:**
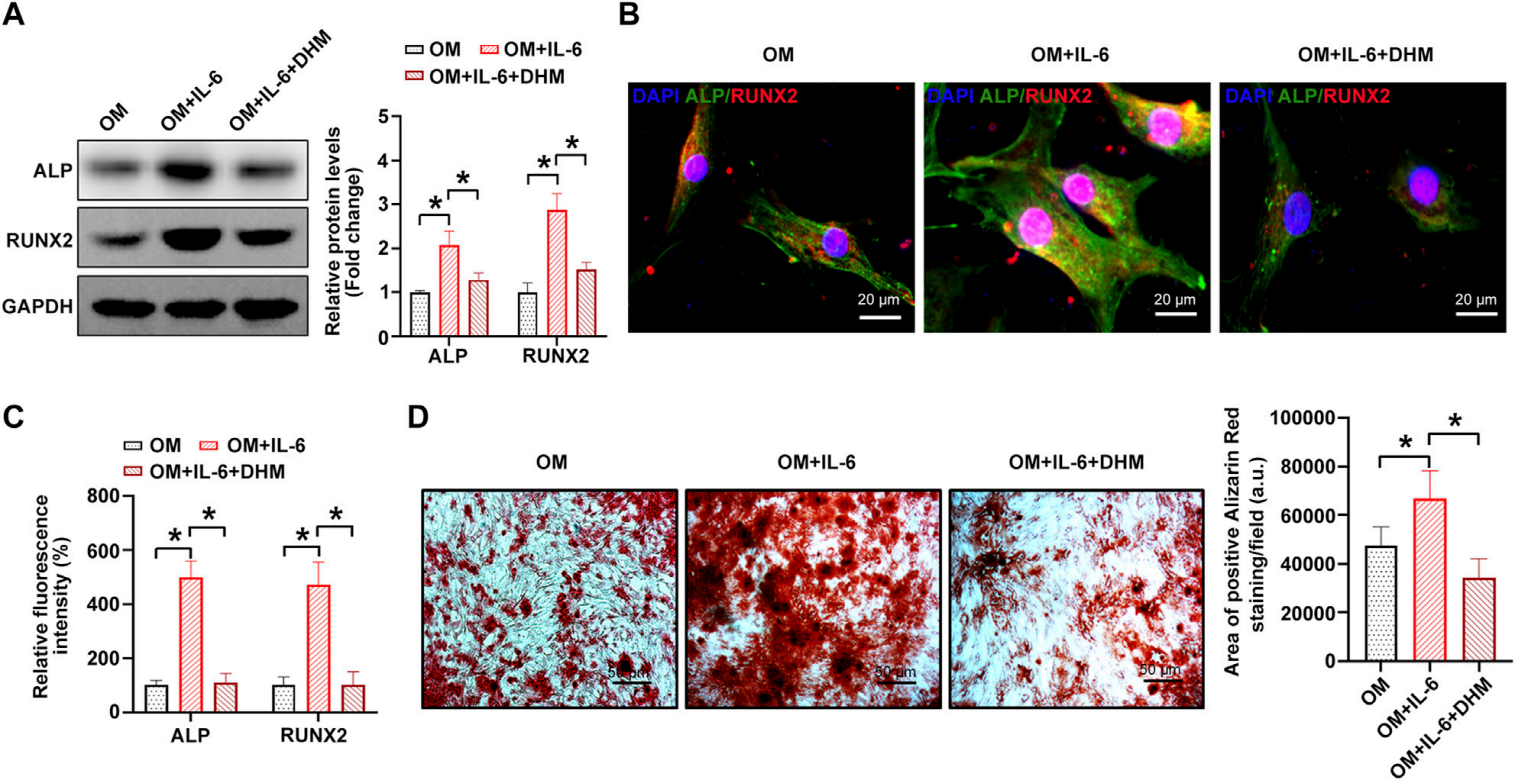
Dihydromyricetin (DHM) rescues the osteogenic differentiation phenotypes induced by interleukin-6 (IL-6) in human valvular interstitial cells (hVICs). Western blotting**(A)** and immunofluorescent staining **(B)** were used to detect the protein levels of osteogenesis-specific genes (alkaline phosphatase, ALP, and runt-related transcription Factor 2, RUNX2) in hVICs stimulated with osteogenic induction medium (OM) and then cotreated with IL-6 or IL-6+DHM. One-way ANOVA followed by Bonferroni *post hoc test*. **(C)** Semiquantification of the fluorescence intensity of ALP and RUNX2. One-way ANOVA followed by Bonferroni *post hoc test*. **(D)** Alizarin red staining of mineralization nodules in hVICs stimulated with OM and then cotreated with IL-6 or IL-6+DHM. One-way ANOVA followed by Bonferroni *post hoc test*. *N* = 3 per group. Values are the mean ± SD. *p < 0.05 indicates a significant difference.

Next, we performed a rescue experiment to confirm whether IL-6 downregulation contributes to the inhibitory effect of DHM on the osteogenic differentiation of hVICs. The results indicate that the procalcific effects of IL-6 were partially reversed by DHM, as evidenced by decreased osteogenesis-specific gene (ALP and RUNX2) expression (Figures 5A–C) and reduced calcium deposition in hVICs (Figure 5D). Collectively, these data suggest that DHM inhibits the osteogenic differentiation of hVICs by downregulating IL-6.

## Discussion

The key pathogenesis of CAVD involves inflammation, lipoprotein deposition, and osteogenic differentiation of hVICs (Aikawa and Libby, 2017). Currently, although a few risk factors have been identified, there are still no approved pharmacotherapies in the clinic for the treatment and prevention of CAVD (Tsimikas, 2019). Hence, there is growing interest in exploring effective pharmacotherapeutic interventions for CAVD. Our current study showed that DHM administration functions as an effective medical therapy for negating the osteogenic differentiation of hVICs. We provide the first evidence that DHM exerts inhibitory effects on IL-6 expression by interacting with c-KIT and thereby blocking OM-induced c-KIT phosphorylation. Notably, c-KIT inhibition markedly reduced AVC progression *in vitro*. Importantly, we also identified IL-6 as a substantial downstream target of DHM for ameliorating the osteogenic differentiation of hVICs. These findings pinpoint a previously unidentified regulatory DHM/c-KIT/IL-6 axis contributing to CAVD, suggesting that DHM might be an effective chemical drug for the treatment of CAVD.

Mounting evidence suggests that DHM exhibits cardiovascular protective properties in cardiovascular diseases, such as vascular calcification (Feng et al., 2021), endothelial protection (Chen et al., 2021), and pulmonary arterial hypertension (Li Q et al., 2017). In atherosclerosis, which shares similar pathological features with CAVD (Kostyunin et al., 2020), DHM has been shown to increase endothelial nitric oxide production in apolipoprotein E-deficient mice (ApoE^−/−^) (Yang et al., 2020) and protect human umbilical vein endothelial cells from oxidative damage (Zhang et al., 2019). Whether DHM exhibits a protective role against CAVD progression in humans requires further study. Our present study demonstrated that DHM prevents the osteogenic differentiation of hVICs *via* a c-KIT/IL-6-dependent pathway at noncytotoxic concentrations of 20 μM, as evidenced by decreased protein levels of two osteogenesis-specific genes (ALP and Runx2) and calcified nodule formation, which is consistent with several previous findings involving the protective role of DHM in atherosclerosis and vascular calcification (Liu et al., 2017; Yang et al., 2020; Feng et al., 2021). In addition, a recent study indicated that DHM significantly suppressed foam cell formation by regulating cholesterol efflux in macrophages (Zeng et al., 2018), thereby protecting ApoE^−/−^ mice from atherosclerosis. Importantly, Matsumoto et al. (2010) reported that endothelial integrity of the aortic valves serves as a crucial cellular mechanism of CAVD progression, and our previous study also revealed that macrophage polarization could regulate AVC progression (Li G et al., 2017). Thus, further investigation is needed to elucidate whether DHM could ameliorate AVC by regulating the pathological processes attributed to endothelial cells and macrophages in the valve district. Importantly, DHM supplementation was shown to exert a strong beneficial effect on improving the glycemic control of type 2 diabetes mellitus (T2DM) patients (Ran et al., 2019), and a previous randomized controlled trial confirmed the therapeutic effects of DHM supplementation in patients with nonalcoholic fatty liver disease (Chen et al., 2015). Thus, DHM supplementation may provide an effective and safe pharmacological strategy for patients suffering from CAVD. However, as we stated, prior to clinical use, randomized clinical trials are still needed to sufficiently validate the safety and therapeutic potential of DHM in CAVD treatment.

Increasing evidence suggests that inflammatory infiltration and subsequent endothelial damage are hallmarks of early lesions in CAVD (Bartoli-Leonard et al., 2021; Lu et al., 2022; Zhang et al., 2022). Inflammatory factors are secreted by several resident cells of the valve layer or inflammatory cells, which in turn can facilitate the process of CAVD. Notably, it was reported that not only valve endothelial cells (Mahler et al., 2013) but also valve interstitial cells (El Husseini et al., 2014) could produce IL-6, which is a strong promoter of CAVD. Several *in vitro* studies have shown that DHM prevents atherosclerosis by reducing IL-6 production (Li Q et al., 2017; Liu et al., 2017). In addition, it has previously been shown that activation of c-KIT could significantly promote IL-6 expression (Das Roy et al., 2013). However, the interactive relationship of DHM, c-KIT, and IL-6 in CAVD remains uncharacterized. Here, we demonstrated for the first time that DHM interacted with c-KIT and thereby ameliorated the osteogenic differentiation of hVICs by inhibiting IL-6 expression. In fact, c-KIT activation could also promote the expression of some other inflammatory factors, such as TNF-α and IL-10 (41). Thus, whether additional inflammatory factors could be downstream targets of the DHM/c-KIT pathway requires further investigation. Interestingly, in a previous study, Song et al. (2016) demonstrated that c-KIT is atheroprotective in hyperlipidemic mice by antagonizing kruppel-like Factor 4, which was supported by some of the authors’ latest studies (Song et al., 2019; Zigmond et al., 2021). However, our present study indicates that the DHM-mediated increase in c-KIT phosphorylation exerts procalcific effects through IL-6 upregulation. We argue that the stage of the disease was an important confounding factor, resulting in this difference.

Only *in vitro* experiments were conducted in the present study to explore the role of DHM and c-KIT in the progression of osteogenic differentiation of hVICs. Thus, whether DHM and c-KIT regulate CAVD progression *in vivo* still requires further investigation using preclinical animal models. Specifically, further *in vivo* studies using c-KIT gene-deficient mice or a murine model harboring a cell-type-specific deletion of c-KIT in valve interstitial cells may provide more compelling evidence. Furthermore, to the best of our knowledge, the pathological mechanisms of bicuspid CAVD are different from those of tricuspid CAVD (Kostina et al., 2018). The results of the present study were only based on hVICs isolated from tricuspid aortic valves (TAVs) rather than bicuspid aortic valves (BAVs) and may only be applied to tricuspid CAVD. Thus, whether DHM also plays a protective role in bicuspid CAVD warrants further investigation.

## Conclusion

**FIGURE 6:**
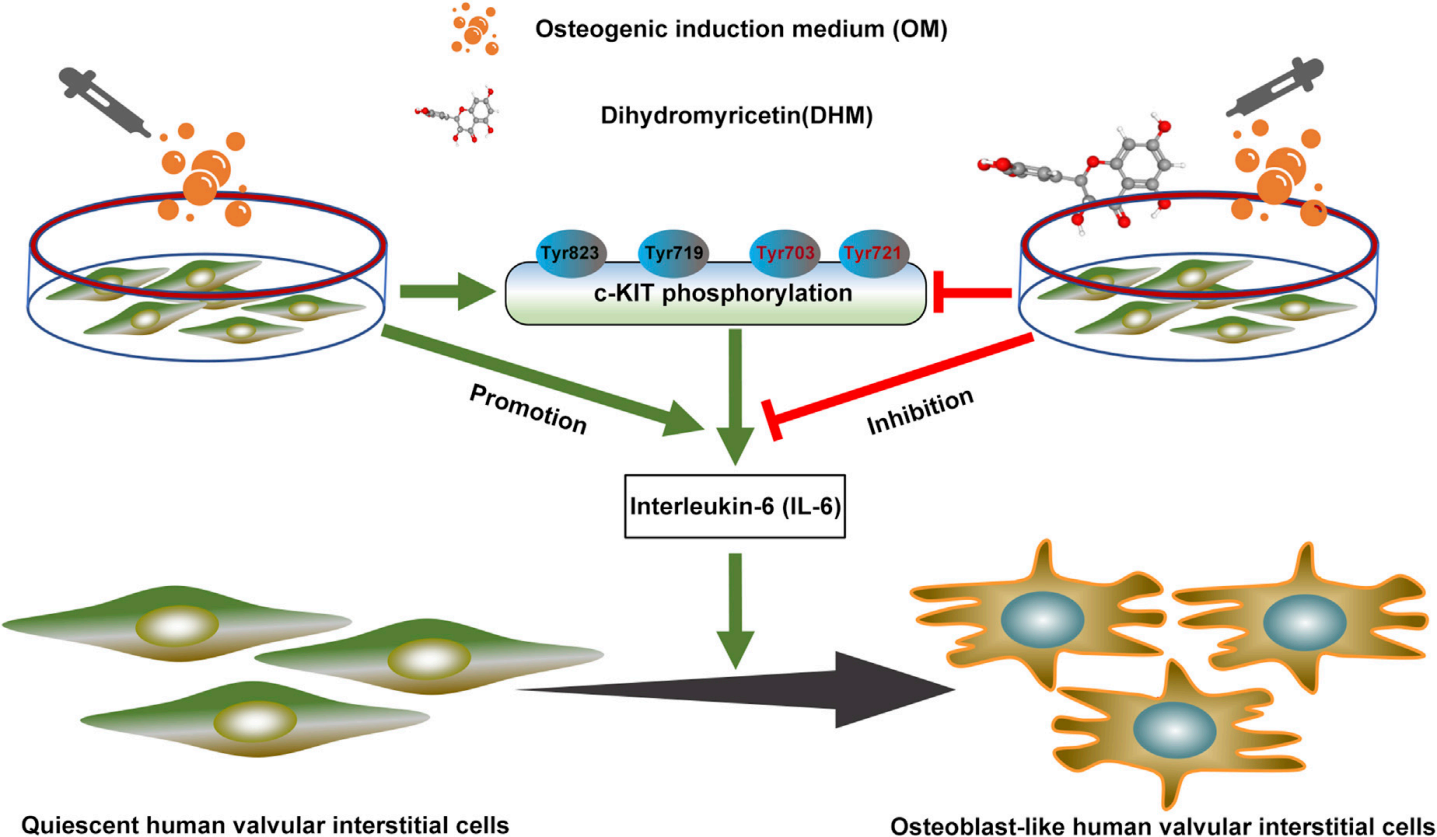
Dihydromyricetin (DHM) downregulated interleukin-6 (IL-6) expression by antagonizing phosphorylation of c-KIT, thereby preventing osteogenic differentiation of human valvular interstitial cells.

In this study, we confirmed that DHM significantly ameliorates the osteogenic differentiation of hVICs. Mechanistically, DHM repressed c-KIT phosphorylation, thereby resulting in IL-6 downregulation in hVICs. These findings pinpoint a previously unidentified DHM/c-KIT/IL-6 axis contributing to the inhibition of osteogenic differentiation of hVICs (Figure 6). Thus, DHM treatment may represent a potent pharmacological remedy to prevent CAVD progression.

## Data Availability

The datasets presented in this study can be found in online repositories. The names of the repository/repositories and accession number(s) can be found in the article/supplementary material.
